# Short-Term Outcome of Patients with Cirrhosis and Concurrent Portal Cavernoma Presenting with Acute Variceal Bleeding

**DOI:** 10.1155/2018/9491856

**Published:** 2018-01-24

**Authors:** Xuefeng Luo, Wanqin Wang, Xiaoli Fan, Ying Zhao, Xiaoze Wang, Jinlin Yang, Li Yang

**Affiliations:** Department of Gastroenterology and Hepatology, West China Hospital, Sichuan University, Chengdu, China

## Abstract

**Background and Aim:**

The outcome of cirrhotic patients with main portal vein occlusion and portal cavernoma after the first episode of acute variceal bleeding (AVB) is unknown. We compared short-term outcomes after AVB in cirrhotic patients with and without portal cavernoma.

**Methods:**

Between January 2009 and September 2014, 28 patients with cirrhosis and portal cavernoma presenting with the first occurrence of AVB and 56 age-, sex-, and Child-Pugh score-matched cirrhotic patients without portal cavernoma were included. The primary endpoints were 5-day treatment failure and 6-week mortality.

**Results:**

The 5-day treatment failure rate was higher in the cavernoma group than in the control group (32.1% versus 12.5%; *p* = 0.031). The 6-week mortality rate did not differ between the cavernoma and control group (25% versus 12.5%, *p* = 0.137). Multivariable Cox proportional hazard regression analyses revealed that 5-day treatment failure (HR = 1.223, 95% CI = 1.082 to 1.384; *p* = 0.001) independently predicted 6-week mortality.

**Conclusions:**

Cirrhotic patients with AVB and portal cavernoma have worse short-term prognosis than patients without portal cavernoma. The 5-day treatment failure was an independent risk factor for 6-week mortality in patients with cirrhosis and portal cavernoma.

## 1. Introduction

Acute variceal bleeding (AVB) remains one of the most dangerous complications of portal hypertension and is associated with significant morbidity and mortality in patients with cirrhosis [1]. Despite the advances in AVB management, 6-week mortality still remains high and is related to the severity of the underlying cirrhosis, hepatic venous pressure gradient (HVPG), and the presence of portal vein thrombosis [1–5].

With the increased frequency of liver imaging modalities, portal vein thrombosis (PVT) is increasingly identified in the setting of liver cirrhosis. The estimated prevalence of PVT in these patients varies from 5 to 26% [6, 7]. The chronic PVT may result in main portal vein occlusion and cavernous transformation, while the obstructed portal vein is replaced by a network of hepatopetal vessels [8]. The presence of portal cavernoma negatively affects the prognosis of these patients because of the resultant further increases in portal hypertension, with an increased risk of variceal bleeding, and, furthermore, the extension of PVT may increase the risk of perioperative morbidity and mortality or exclude patients from liver transplantation [9, 10].

The effect of main portal vein occlusion and cavernous transformation on the short-term outcomes of patients with cirrhosis and AVB has not been previously described. The aim of the present study was to evaluate the short-term outcomes of patients with cirrhosis and concurrent main portal vein occlusion and portal cavernoma admitted due to the first episode of AVB in a retrospective matched study.

## 2. Materials and Methods

### 2.1. Study Cohort

This retrospective cohort study included consecutive patients with cirrhosis, admitted to West China Hospital (a 4800-bed tertiary medical center in China) from January 2009 to September 2014, with the first episode of AVB and portal cavernoma. Liver cirrhosis was diagnosed by liver biopsy and/or clinical and imaging findings. For each identified patient, 2 patients with cirrhosis and the first episode of AVB without the presence of PVT, admitted during the same period, were matched for age, sex, and Child-Pugh score. Patients with hepatocellular carcinoma were excluded. This study was approved by the Institutional Review Board of West China Hospital.

### 2.2. Treatments

Vasoactive drugs (somatostatin) were administered to every patient as soon as possible after gastrointestinal bleeding and before endoscopic examination. Endoscopic treatment was performed in patients diagnosed with bleeding from gastroesophageal varices on endoscopy. Endoscopic variceal banding and endoscopic glue injection were used as the primary therapies for bleeding from esophageal or gastric varices, respectively.

### 2.3. Data Collection and Definition

Complete baseline data were collected through electronic medical records. These data included demographic information, etiology of liver cirrhosis, and basic laboratory information including regular blood tests, hepatic and renal function tests, prothrombin time, international normalized ratio (INR), Child-Pugh score and Model for End-Stage Liver Diseases (MELD) scores, imaging findings on color Doppler ultrasound, contrast-enhanced computed tomography (CT) and magnetic resonance imaging (MRI), and information about the treatments, including endoscopic therapy, surgery, and use of *β*-blockers.

The diagnosis of portal cavernoma was based on contrast-enhanced CT or MRI, in which the main portal vein was completely obstructed and replaced by fibrous tissue with the development of a hepatopetal network of periportal collateral veins. The time frame for an acute bleeding episode was 120 h (5 days). Five-day treatment failure was defined as uncontrolled index variceal bleeding, rebleeding, or death within 5 days. In particular, we defined the control of active variceal bleeding as a lack of hematemesis; the hemoglobin (Hb) level was stable without requiring blood transfusions or hemodynamic stability for 24 h after drug therapy and endoscopic treatment. Early rebleeding was defined by any of the following events, whichever occurred first: fresh hematemesis or NG aspiration of 100 ml of fresh blood 2 h after the start of vasoactive drugs alone or in combination with endoscopic therapy; development of hypovolemic shock; or a 3 g decrease in Hb (9% drop of Ht) within any 24 h period if no transfusion is administered [11].

### 2.4. Statistical Analysis

Quantitative variables were expressed as the mean ± standard deviation, and Student's* t*-test was performed to assess the difference between variables. Qualitative variables were expressed as frequencies and were analyzed using Pearson's *χ*2 test or Fisher's exact test. The cumulative incidence of 6-week mortality rate was estimated using the Kaplan–Meier method and compared using the log-rank test. Independent predictors with a *p* value < 0.05 in univariate analysis were included in the multivariate analyses. Multivariate analyses were performed using Cox regression analysis. Individual variables already included in the Child-Pugh score or MELD score were not considered separately. The risks estimated from the Cox regression models were expressed as hazard ratios (HRs) with their respective 95% confidence intervals (CIs). A two-tailed *p* value of <0.05 was considered statistically significant. Statistical analysis was performed using SPSS for windows (version 19.0; SPSS, Chicago, IL).

## 3. Results

### 3.1. Characteristics of the Cohorts

Twenty-eight patients with cirrhosis and concurrent portal cavernoma presenting with the first episode of AVB were admitted in our hospital during the period of the study. Fifty-six age-, sex-, and Child-Pugh score-matched patients with cirrhosis who experienced the first incident of AVB without PVT were selected from the overall population of patients during the same time period. Overall, a total of 84 patients were enrolled in the study.

The clinical and laboratory characteristics of the cohort are summarized in Table 1. The most common etiology for liver cirrhosis was Hepatitis B Virus infection (60%) followed by alcoholic liver disease (13%). The mean Child-Pugh and MELD score at the time of hospital admission were 8.1 ± 1.9 and 15.8 ± 5.4, respectively. Bleeding originated from esophageal varices in 22 patients (78.6%), from gastric varices in 2 patients (7.1%), and from unknown site in 4 (14.3%) in the cavernoma group. Source of bleeding was esophageal varices in 40 patients (71.4%), gastric varices in 10 patients (17.9%), and unknown in 6 (10.7%) in the control group. These two groups had comparable clinical and laboratory data, except for the higher platelet level, more incidences of ascites and previous splenectomy in the cavernoma group.

### 3.2. 5-Day Treatment Failure

Table 2 shows the main outcomes of the cohort of patients. Failure to control the index-bleeding episode occurred in 5 cases in the cavernoma group and in 3 cases in the control group. The frequency of index bleeding was not controlled successfully by initial therapy and did not differ significantly between the cavernoma group and the control group (17.9% versus 5.4%, *p* = 0.066). In the cavernoma group, 2 patients were treated with balloon tamponade as a rescue therapy. One patient died of massive bleeding before further intervention could be performed, and 2 patients received pharmacological therapy alone. All 3 patients in the control group underwent transjugular intrahepatic portosystemic shunt (TIPS) as a salvage therapy.

Of the 67 patients in whom index bleeding was controlled successfully without rescue therapy, 6 (9.0%) patients rebled from varices within the first 5 days of their hospital admission. However, the rate of recurrent variceal bleeding did not differ significantly between these two groups (14.3% versus 3.6%, *p* = 0.072). Five patients (3 in the cavernoma group and 2 in the control group) died within the 5-day treatment period.

Ultimately, 5-day treatment failure occurred in 9 out of 28 patients (32.1%) in the cavernoma group and in 7 out of 56 patients (12.5%) in the control group. The rate of 5-day treatment failure was higher in the cavernoma group compared with the control group (*p* = 0.031). Among all of the subjects, no significant independent predictor of 5-day treatment failure was identified using univariate regression and Cox proportional hazard modeling.

### 3.3. 6-Week Mortality

**Figure 1:**
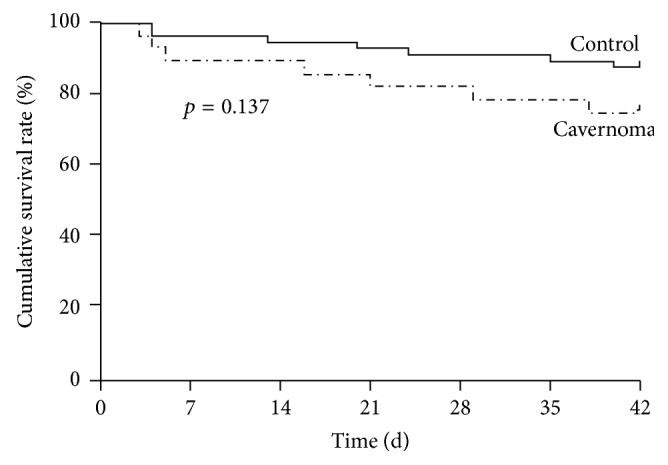
Kaplan–Meier estimates of 6-week survival in cirrhotic patients with portal cavernoma and active variceal bleeding.

The overall 6-week mortality after AVB was 16.7% (*n* = 14) in the entire series, with 35.7% (*n* = 5) occurring within five days. The cumulative mortality rate did not differ between the cavernoma and control group (25% versus 12.5%, *p* = 0.137, Figure 1). Causes of death were uncontrolled or reported as relapsing bleeding (*n* = 7), liver failure (*n* = 5), sepsis (*n* = 1), and unknown (*n* = 1). The multivariable Cox proportional hazard regression analysis identified 5-day treatment failure (HR = 1.223, 95% CI = 1.082 to 1.384; *p* = 0.001) as the only independent predictor of 6-week mortality.

## 4. Discussion

Hemorrhage from gastroesophageal varices represents one of the most feared complications in the natural history of cirrhosis and is associated with significant morbidity and mortality [1, 3]. Our study was specifically designed to assess the short-term outcomes of cirrhotic patients with concurrent main portal vein occlusion and portal cavernoma presenting with the first episode of AVB. We found that the presence of portal cavernoma was associated with worse 5-day treatment failure in these patients.

The results of our study confirmed that PVT increased the risk of 5-day treatment failure in patients with AVB [2, 12]. In 2003, D'Amico and de Franchis evaluated the short-term outcomes and risk factors of 185 patients with liver cirrhosis and AVB retrospectively. They reported that the presence of PVT was an independent predictor for 5-day failure (for any sources of bleeding: OR = 3.19, 95% CI: 1.53–6.67, *p* = 0.002; for variceal bleeding: OR = 3.06, 95% CI: 1.39–6.68, *p* = 0.005) [2]. Another Italian study performed in 2012 enrolled 185 patients of whom 32 patients (17.3%) had PVT and demonstrated that the presence of PVT was associated with negative short-term outcomes [12]. This finding was potentially attributed to the higher portal venous pressure due to main portal vein occlusion, which makes the patients more susceptible to both initial medical/endoscopic therapy failure and early rebleeding. However, liver perfusion was decreased to a great extent, and further hepatic decompensation may occur.

TIPS used to be considered as a rescue treatment when bleeding was not controlled by less invasive endoscopic treatment and/or pharmacological therapy [3]. However, there is growing evidence for the support of early TIPS within 72 h (ideally < 24 h) in selected patients at a high risk of treatment failure [e.g., HVPG ≥ 20 mmHg, Child-Pugh C < 14 points, or Child-Pugh B with active bleeding] [13, 14]. All 3 patients who failed the initial therapy in the control group underwent TIPS as a rescue treatment and survived. In contrast, none of the patients in the cavernoma group could benefit from early placement of TIPS due to the complete obstruction of the main portal vein and lack of larger portal-portal collateral vein. The limitations of the treatment options demonstrated negative effects on the outcomes of the patient.

Currently, the effect of PVT on the prognosis of cirrhosis is debatable [7]. Luca et al., in their series of 42 patients with cirrhosis and partial PVT, revealed that liver function at diagnosis was the only independent predictor of survival and hepatic decompensation, instead of progression or regression of PVT [15]. Similarly, Maruyama and his colleagues found that the incidence of variceal bleeding did not differ statistically between patients with PVT compared to patients without PVT, as well as the survival rate [16]. Finally, a very recent prospective study, performed by Nery et al., enrolled 1,243 adults (863 Child A patients and 380 Child B patients) with cirrhosis but without PVT [17]. They found that the 5-year cumulative incidence of PVT was 10.7%, and PVT was mostly partial and varied overtime. In addition, there was no relationship between the development of PVT and the progression of liver disease, which was consistent with the findings obtained in previous reports [17].

Importantly, whether PVT affects the treatment effect and patients' survival in cirrhosis is associated with the degree and extension of PVT, as well as the severity of the underlying liver disease. In a study by Luca et al., which included 42 patients with cirrhosis and PVT, only 9 patients (21.4%) had grade 4 (≥76% of vessel lumen) thrombosis, 5 (11.9%) patients had large esophageal varices, and 12 (28.6%) patients experienced gastroesophageal variceal bleeding [15]. Over the 11-year study period, PVT developed in 28% (42/150) of patients with cirrhosis, and 11 patients (26.2%) suffered from complete portal vein obstruction [16]. Furthermore, 183 out of 1243 patients (16.3%) had grade ≥2 esophageal varices in a study by Nery et al. [17]. In the present series, all patients in the cavernoma group were admitted due to AVB. Unlike previous reports with a relatively high rate of spontaneous thrombosis improvement (45–47.5%), the possibility of spontaneous recanalization of the occluded main portal vein is very low [15, 16]. In addition, the mean MELD score (15.8) of our cohorts appeared to be higher than previous reports (12.1 from Luca, 10.2 in patients without PVT, and 10.6 in patients with PVT from Maruyama, resp.).

To the best of our knowledge, this is the first study to focus on the effect of portal cavernoma on the short-term outcomes of patients with cirrhosis and the first episode of AVB. The optimal management of AVB in patients with cirrhosis and portal cavernoma was difficult to establish due to the scarcity of the clinical trial data. This group of patients is usually excluded from clinical trials, and bleeding is particularly difficult to control because TIPS is often not applicable.

The major limitation of our study is the relatively small sample size and its retrospective design; therefore, bias may exist. The definition of portal cavernoma varied between different studies. Only patients with complete occluded main portal vein and the presence of portal-portal collateral vessels were included in the present study which influenced the number of patients for analysis greatly. We are aware that a prospective, large sample size trial may provide more convincing evidence on this subject. Unfortunately, only 28 patients with cirrhosis and portal cavernoma who presented with the first episode of AVB were identified during the study period in our hospital, which is one of the largest single-site hospitals in the world.

In conclusion, our data indicate that portal cavernoma in cirrhotic patients suffering from the first episode of AVB is associated with higher 5-day treatment failure. However, 6-week mortality did not significantly differ between the cavernoma and control groups. Long-term outcomes of patients with cirrhosis and portal cavernoma need further evaluation.

## Figures and Tables

**Table 1:** Demographics and clinical characteristics at admission.

Characteristic	The cavernoma group (*n* = 28)	The control group (*n* = 56)	*p* value
Age, years	53.1 ± 11.9	53.3 ± 11.8	0.487
Sex, male, %	19/9	38/18	1
Cause of liver disease, *n*			0.580
Chronic HBV infection	19	31	
Chronic HCV infection	0	2	
Alcohol	3	9	
others	6	14	
Hemoglobin, g/L	79.6 ± 23.9	73.2 ± 23.5	0.242
Platelet, 1000/mm3	121.7 ± 101.9	84.7 ± 57.1	0.036
WBC	5.3 ± 3.1	9.1 ± 11.3	0.091
Creatinine, *µ*mol/L	70.0 ± 20.4	76.4 ± 20.9	0.962
ALT, IU/L	30.3 ± 19.7	41.0 ± 56.2	0.330
AST, IU/L	48.6 ± 30.3	57.2 ± 92.9	0.633
Bilirubin, *µ*mol/L	35.9 ± 38.3	26.1 ± 22.9	0.149
Albumin, g/L	29.7 ± 7.0	30.5 ± 5.8	0.554
INR	1.3 ± 0.4	1.4 ± 0.4	0.455
Ascites, *n* (%)	24 (85.7%)	33 (58.9%)	0.013
Encephalopathy, *n* (%)	1 (3.6%)	1 (1.7%)	0.613
Child-Pugh grade, *n* (%)			0.447
A	4	14	
B	18	34	
C	6	8	
Child score	7.36 ± 2.14	7.96 ± 1.76	0.439
MELD score	15.43 ± 5.3	16.46 ± 5.4	0.851
Heart rate, beats/min	84 ± 13	83 ± 15	0.983
Systolic blood pressure, mmHg	109 ± 20	112 ± 18	0.530
Infection, *n* (%)	7 (25%)	12 (20.7%)	0.712
Splenectomy, *n* (%)	10 (35.7%)	9 (15.5%)	0.043

*Abbreviations*. HBV, hepatitis B virus; HCV, hepatitis C virus; WBC, white blood cell; ALT, alanine aminotransferase; AST, aspartate aminotransferase.

**Table 2:** Summary of efficacy measurements during the 5-day period.

	The cavernoma group (*n* = 28)	The control group (*n* = 56)	*p* value
5-day treatment failure, *n* (%)	9 (32.1%)	7 (12.5%)	0.031
Failure to control acute bleeding, *n* (%)	5 (17.9%)	3 (5.4%)	0.066
Early rebleeding in 5 days, *n* (%)	4 (14.3%)	2 (3.6%)	0.072
5-day mortality, *n* (%)	3 (10.7%)	2 (3.6%)	0.192
